# Deep Sequencing of RNA from Three Different Extracellular Vesicle (EV) Subtypes Released from the Human LIM1863 Colon Cancer Cell Line Uncovers Distinct Mirna-Enrichment Signatures

**DOI:** 10.1371/journal.pone.0110314

**Published:** 2014-10-17

**Authors:** Hong Ji, Maoshan Chen, David W. Greening, Weifeng He, Alin Rai, Wenwei Zhang, Richard J. Simpson

**Affiliations:** 1 La Trobe Institute for Molecular Science (LIMS), La Trobe University, Melbourne, Victoria, Australia; 2 Chongqing Key Laboratory for Disease proteomics; and State Key Laboratory of Trauma, Burns and Combined Injury, Institute of Burn Research, Southwest Hospital, Third Military Medical University, Chongqing, China; 3 BGI-Shenzhen, Shenzhen, China; University of California, San Diego, United States of America

## Abstract

Secreted microRNAs (miRNAs) enclosed within extracellular vesicles (EVs) play a pivotal role in intercellular communication by regulating recipient cell gene expression and affecting target cell function. Here, we report the isolation of three distinct EV subtypes from the human colon carcinoma cell line LIM1863 – shed microvesicles (sMVs) and two exosome populations (immunoaffinity isolated A33-exosomes and EpCAM-exosomes). Deep sequencing of miRNA libraries prepared from parental LIM1863 cells/derived EV subtype RNA yielded 254 miRNA identifications, of which 63 are selectively enriched in the EVs - miR-19a/b-3p, miR-378a/c/d, and miR-577 and members of the let-7 and miR-8 families being the most prominent. Let-7a-3p*, let-7f-1-3p*, miR-451a, miR-574-5p*, miR-4454 and miR-7641 are common to all EV subtypes, and 6 miRNAs (miR-320a/b/c/d, miR-221-3p, and miR-200c-3p) discern LIM1863 exosomes from sMVs; miR-98-5p was selectively represented only in sMVs. Notably, A33-Exos contained the largest number (32) of exclusively-enriched miRNAs; 14 of these miRNAs have not been reported in the context of CRC tissue/biofluid analyses and warrant further examination as potential diagnostic markers of CRC. Surprisingly, miRNA passenger strands (star miRNAs) for miR-3613-3p*, -362-3p*, -625-3p*, -6842-3p* were the dominant strand in A33-Exos, the converse to that observed in parental cells. This finding suggests miRNA biogenesis may be interlinked with endosomal/exosomal processing.

## Introduction

Extracellular vesicles (EVs) are nano-membranous particles ranging from 30–2,000 nm in diameter that are released from most cell types into the extracellular environment [1]. EVs are thought to comprise three main classes depending on their origin – exosomes (Exos, 50–150 nm), shed microvesicles (sMVs, 400–1,500 nm), and apoptotic bodies (400–2,500 nm). Although there is an ongoing polemic amongst researchers regarding the nomenclature, biogenesis, biochemical and functional properties of EV subtypes, the available evidence suggest that exosomes originate by the inward budding of endosomal compartments called multivesicular bodies (MVBs) and are released from the cell into the microenvironment following fusion of MVBs with the plasma membrane, sMVs (ectosomes, microvesicles, microparticles, oncosomes) by outward budding/blebbing from the plasma membrane, and apoptotic bodies through the process of apoptosis/cell shrinkage/nuclear fragmentation [2]. At both functional and biochemical levels, exosomes have been the most widely studied of the EVs. Exosomes have been shown to contain diverse proteins (including oncoproteins, tumour suppressor proteins, transcriptional regulators, splicing factors [1], [3], [4], [5], [6], lipids [7], and RNAs (mRNAs, microRNAs (miRNAs) and other non-coding RNAs) [8] – exosomal molecular cargo information can be accessed by publically-accessible databases such as ExoCarta [9] and EVPedia [10]. Although long regarded as cellular debris, recent exosome studies demonstrate that they have important biological roles in the immune, cardiovascular, and nervous systems and in the pathogenesis of diseases such as cancer [11], [12], [13]. In the last decade it has been established that EVs play a pivotal role in cancer progression and pre-metastatic niche priming for tumour engraftment [14], [15], [16], [17].

It is well recognized that the tumour microenvironment plays a critical role in cancer initiation, progression and metastasis [18]. Intercellular communication between tumour-stroma can be mediated by soluble factors, including cytokines, chemokines, and growth factors [19]. An emerging concept is that tumour-stroma interactions can also involve the direct exchange of genetic information, mainly in the form of miRNAs, a class of noncoding RNAs (18–25 nucleotides in length) that regulate the expression of multiple target genes by binding to their encoded mRNAs [13], [20], [21]. This transfer of genetic material can occur when EVs containing miRNA cargo are released by a donor cell into the extracellular environment and are functionally transferred to recipient cells. Transferred miRNAs can be functional both *in vitro* [8], [22], [23], [24], [25], and *in vivo* [26], [27], [28]. Studies have begun to examine the association of microRNA-related polymorphisms and their association with cancer incidence and prognosis as well as the potential for circulating microRNAs or faecal microRNA expression as non-invasive early detection biomarkers for colorectal cancer [29], and utility of miRNAs in recurrence, metastasis and therapeutic outcomes [30]. A major advance in our understanding of exosomal miRNA biology was the finding that sumoylated hnRNPA2B1 directs the loading of certain miRNAs into exosomes through recognition of specific short motifs present in miRNAs [31].

Recently, we described the isolation of two populations of exosomes as well as sMVs from the same human colon carcinoma cell line LIM1863 [4]. The sMVs were prepared by differential centrifugation and exosomes purified by sequential immunocapture using anti-A33- and anti-EpCAM coupled magnetic beads. While the exosome populations (A33-Exos and EpCAM-Exos) could not be distinguished using electron microscopy, buoyant density or stereotypical exosomes markers (TSG101, Alix and HSP70), protein typing using GeLC-MS/MS [3], [6] revealed that their protein compositions were quite distinct – EpCAM-Exos containing classical apical trafficking components and A33-Exos, enriched with basolateral trafficking molecules. The proteome profiles of both exosome populations, in turn, were quite distinct from the initial report of sMVs released into the same culture medium. In order to further define these EV subtypes, we investigated their molecular composition using another *omics* approach, RNA typing.

In this study, we show using deep sequencing that there are a total of 254 miRNAs identified in the four miRNA libraries prepared (A33-Exos, EpCAM-Exos, sMVs and parent LIM1863 cells), of which 63 are highly enriched in EVs. The three LIM1863-derived EV subtypes are enriched with specific miRNAs signatures, when compared with the parental cell line LIM1863. In particular, we report that 32, 2 and 4 miRNAs that are exclusively enriched in A33-Exos, EpCAM-Exos, and sMVs, respectively – some of which enable exosomes to be distinguished from sMVs. Of the 32 miRNAs selectively enriched in A33-Exos, 13 have not been previously implicated with colorectal cancer (CRC) and we discuss how this information can be utilized towards the potential for CRC diagnostics. A notable finding in our study was the finding of ‘passenger strand’ miRNA (miRNA star) sequences enriched in EVs compared to parent LIM1863 cells.

## Materials and Methods

### Cell culture and isolation of extracellular vesicles

LIM1863 cells [32] were initially cultured to ∼80% confluence in a 175-cm^2^ flask in RPMI-1640 medium (Invitrogen, Carlsbad, CA) supplemented with 5% foetal calf serum (FCS), 0.1% insulin-transferrin-selenium (ITS, Invitrogen), 100 U/ml penicillin and 100 µg/ml streptomycin at 37°C and 5% CO_2_. LIM1863 cells (∼3×10^7^ cells) were harvested (140 *g*, 3 min), suspended in 15 ml phenol red free RPMI-1640 medium (containing 0.5% ITS, 100 U/ml penicillin and 100 µg/ml streptomycin) and transferred into the Cultivation chamber of a CELLine CL-1000 Bioreactor classic flask (Integra Biosciences); the Nutrient Supply chamber contained 500 ml of RPMI-1640 supplemented with 5% FCS, 100 U/ml penicillin and 100 µg/ml streptomycin. Cells were cultured at 37°C and 5% CO_2_ atmosphere. Culture medium in Nutrient Supply chamber was replaced twice a week and the cell suspension from the Cultivation chamber was harvested every 48 h. After each collection, the cell suspension was centrifuged at 140 *g* for 3 min to sediment LIM1863 cell organoids, which were resuspended in 15 ml of cultivation medium and re-seeded back into the Cultivation chamber. The supernatant was centrifuged at 2,000 *g* for 10 min to remove floating cells/cell debris and then centrifuged further at 10,000 *g* for 30 min at 4°C to collect shed microvesicles (sMVs). The resulting supernatant was further centrifuged (100,000 *g*, 1 h) to collect crude exosomes. Crude exosomes were fractionated into two distinct exosome subpopulations (A33-Exos and EpCAM-Exos) by sequential immunocapture using Dynabeads (Invitrogen) loaded with anti-human-A33 monoclonal antibodies [33] in tandem with anti-EpCAM (CD326)-antibody bound magnetic microbeads (Miltenyi Biotec), as described [4].

### Protein quantitation

Protein content was estimated by 1D-SDS-PAGE/SYPRO Ruby protein staining densitometry, as previously described [5].

### Transmission electron microscopy (TEM)

A33-Exos and EpCAM-Exos were eluted from their respective magnetic beads with 0.2 M Glycine, pH 2.5 and harvested by centrifugation (100,000 *g*, 1 h). For TEM, samples (sMVs, A33- and EpCAM-Exos, 1 µg/10 µl PBS) were applied for 2 min to 400 mesh copper grids coated with a thin layer of carbon. Excess material was removed by blotting with filter paper, and samples negatively stained twice with 10 µl of a 2% uranyl acetate solution for 10 min (ProSciTech, Queensland, Australia). Grids were air dried and imaged using a JEOL JEM-2010 transmission electron microscope operated at 80 kV.

### Western Blot Analysis

Sample protein concentrations were determined using one-dimensional SDS-PAGE/SYPRO Ruby protein staining/densitometry, as described [5], [6]. Briefly, samples were lysed in SDS sample buffer for 20 min at room temperature, and proteins (10 µg/sample) resolved by SDS-PAGE, electrotransferred onto nitrocellulose membranes using the iBlot Dry Blotting System (Life Technologies), and membranes blocked with 5% (w/v) skim milk powder in Tris-buffered saline containing 0.05% (v/v) Tween-20 (TTBS) for 30 min. Membranes were probed overnight in TTBS with primary mouse anti-CD9 (at 1∶1000) and mouse anti-TSG101 (at 1∶1,000) from BD Biosciences, mouse anti-Alix (at 1∶1,000) from Cell Signalling, or mouse anti-A33 (1 µg/ml) (gift from Dr A Scott, Ludwig Institute for Cancer Research, Melbourne). After washing with TTBS (3×10 min) membranes were incubated with horse radish peroxidase (HRP)-conjugated anti-mouse IgG (Sigma) or IRDye 800 anti-mouse IgG (Li-COR Biosciences). Proteins were visualized by incubating membranes with Western HRP substrate (Merck-Millipore) followed by imaging with ChemiDocMP System (Bio-Rad) or by imaging directly with the Odyssey Infrared Imaging System (v3).

### Total RNA isolation

Total RNA from LIM1863 cells, sMVs, A33- and EpCAM-Exos was isolated with TRIzol (Life Technology), according to manufacturer’s instructions. Briefly, samples were lysed in 1 ml TRIzol Reagent by repetitive pipetting for 5 min at room temperature (RT). Chloroform (0.2 ml/ml TRizol Reagent) was added to solubilized samples and mixtures vortexed vigorously for 15 s, incubated at RT for 2–3 min and then centrifuged (12,000 *g*, 15 min, 4°C). Aqueous phase was collected, mixed with 5 µg of glycogen (20 mg/ml aqueous glycogen, Invitrogen) and isopropyl alcohol (0.5 ml isopropyl alcohol/1 ml aqueous phase) and incubated for 10 min at RT. Total RNA was recovered by centrifugation at 12,000 g for 10 min at 4°C. Resultant RNA pellets were washed once with 75% aqueous ethanol, air-dried for 5 min and re-dissolved in RNase-free water. The quantity, quality and composition of RNA samples were evaluated using an Agilent 2100 Bioanalyzer (Agilent Technologies, USA).

### Small RNA library construction and sequencing

Four small RNA libraries (from parental LIM1863 cells and derived sMVs, A33-, and EpCAM-Exos) were constructed and sequenced with Illumina TruSeq deep sequencing technology (Sample Preparation Guide, Par #15004197 Rev.A, Illumina, San Diego, CA). Briefly, total RNA samples were fractionated on a 15% Tris-borate-EDTA (TBE) polyacrylamide gel (Invitrogen) and a bands corresponding to small RNAs (18∼30 nt) were excised and small RNAs extracted by centrifugation. After ligation of 5′(5′-GUUCAGAGUUCUACAGUCCGACGAUC-3′) and 3′(5′-UGGAAUUCUCGGGUGCCAAGG-3′) adaptors, small RNA molecules were reverse transcribed into cDNA, then amplified using the adaptor primers for 14 cycles and the fragments (∼150 bps) were isolated from a 6% TBE PAGE-gel. The purified cDNA was directly used for cluster generation and sequenced using an Illumina HiSeq 2000 platform. Image files generated by the sequencer were processed to produce digital-quality data (raw FASTQ files). FASTQ files for all four small RNA libraries (LIM1863 cells, and derived sMVs, A33-Exos and EpCAM-Exos) have been submitted to Sequence Read Archive (SRA) of NCBI under the accession number SRA106214.

### Quantitative real-time PCR

Total RNA (3 µl containing 12 ng RNA/µl) prepared from LIM1863 cells and derived EVs was reverse transcribed using a TaqMan miRNA Reverse Transcription (RT) Kit from Applied Biosystems/Life Technologies with Megaplex RT Primers (Human Pool A and Pool B, Applied Biosystems). RT reaction conditions, based on manufacturer’s instructions, were: 40 cycles of 16°C for 2 min, 42°C for 1 min and 50°C for 1 s. Resultant Megaplex RT products (2.5 µl) were then mixed with 22.5 µL of Megaplex PreAmp Reaction Mix containing 2.5 ul Megaplex PreAmp Primers Pool A and B (Applied Biosystems). Pre-amplification cycling conditions were: 95°C for 10 min, 55°C for 2 min, 75°C for 2 min followed by 12 cycles of 95°C for 15 s and 60°C for 4 min. After diluting the pre-amplified cDNAs (25 µl) with 75 µl of Tris-EDTA buffer (1 mM Tris buffer containing 0.1 mM EDTA, pH 8.0), 15 µl of diluted cDNA product was mixed with 450 µl of TaqMan Universal PCR Master Mix (Applied Biosystems) and 435 µl nuclease-free water. To validate the deep sequencing data the mixture was subjected to quantitative real-time PCR (qRT-PCR) analysis using TaqMan low-density array (TLDA) cards (set v3.0) representing a total of 754 assays specific to human miRNAs (present in Sanger miRBase v14). qRT-PCR was performed on a 7900 HT Thermocycler (Applied Biosystems) using the manufacturer’s recommended cycling conditions: 50°C for 2 min, 95°C for 10 min followed by 40 cycles of 95°C for 15 s and 60°C for 1 min. qRT-PCR data was collected at the end of each cycle. Cycle threshold (Ct) values were calculated using the SDS software v2.4; automatic baseline settings were assigned a minimum Ct threshold value of 0.2. Ct values >35 were considered to be below assay detection level and excluded from data analysis. Data was analysed using the ΔΔCt method with LIM1863 cell RNA as the reference and global normalization with U6 snRNA and MaMMU6 as candidate controls using Expression Suit Software v1.0.3 (Applied Biosystems).

### Bioinformatic analyses

A bioinformatics pipeline developed in BGI-Shenzhen was employed to identify miRNAs and other small RNA categories. Briefly, low-quality reads (small RNAs which contain base “N” (undefined by the sequencer), or more than 6 bases with quality lower than 13, or more than 4 bases with quality lower than 10), adaptors and reads smaller than 18 nt were excluded from the raw data to generate clean reads (18–30 nt). Clean reads were aligned to miRBase (v20, http://www.mirbase.org) using BLAST software from NCBI to identify known miRNAs and generate expression profiles. Fold changes in expression levels (sample group versus control) were calculated for each miRNA as log_2_ ratios using normalized TPM (transcripts per million reads) values according to the formula: Fold change = log_2_ (sample group/control). P-value for each miRNA in one pairwise comparison was performed based on the Poisson test [34]. Clean reads were aligned to the Human Reference Genome (hg19, http://hgdownload.soe.ucsc.edu/) using SOAP 2 [35] to classify repeat associate small RNAs and mRNA degraded fragments. rRNA, tRNA, snRNA, snoRNA, srpRNA were identified by mapping clean reads to GenBank (NCBI, http://www.ncbi.nlm.nih.gov/) and Rfam (http://rfam.sanger.ac.uk/) databases.

### Gene Ontology and KEGG Pathway Analysis

TargetScan 6.0 (http://www.targetscan.org) was employed to predict target genes for the miRNA candidates. The potential functions of miRNA target genes were annotated by Gene Ontology and KEGG pathway database. WEGO method [36] was used to present significant GO terms. Two statistical values, *P-value* (Fisher’s exact test) and *q-value* [37], were calculated to obtain pathways that were significantly enriched and control the false discovery rate.

## Results

### Presence of RNA in EVs released from the human colon carcinoma cell line LIM1863

**Figure 1:**
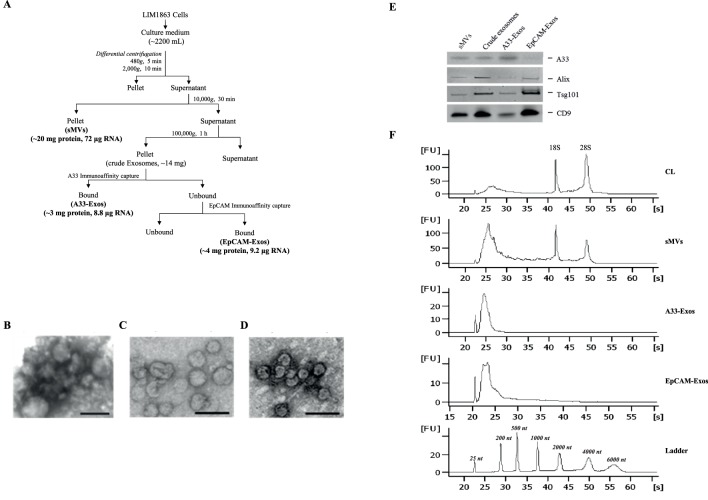
Human colon carcinoma cell line LIM1863 release two populations of exosomes (A33-Exos and EpCAM-Exos) and shed microvesicles (sMVs). **(A)** LIM1863 cells were grown in RPMI-1640 medium supplemented with 5% FCS (exosome-depleted), 100 U/ml penicillin and 100 µg/ml streptomycin in CELLine Bioreactor classic flasks and the culture medium (CM) collected. sMVs were first isolated from the CM (yield: ∼20 mg protein, 72 µg RNA). Next, A33-Exos were isolated from the sMV-free CM via anti-A33 antibody capture (yield: ∼3 mg protein, 8.8 µg RNA) and EpCAM-Exos were isolated from the A33-Exos-depleted CM using EpCAM-coupled magnetic beads (yield: ∼3 mg protein, 9.2 µg RNA). **(B-D)** Electron microscopy images of sMVs **(B)**, A33-Exos **(C)** and EpCAM-Exos **(D)** showing a size of 150–300 nm diameter and 40–100 nm diameter for sMVs and A33−/EpCAM-Exos, respectively. Scale bar: 100 nm (n = 3). **(E)** Western blot of EVs (10 µg protein) for A33, Alix (PDCD6IP), TSG101, and CD9. **(F)** Total RNA electropherogram analysis (Agilent Bioanalyzer) from LIM1863 cells and derived EVs. Y axis of electropherogram is arbitrary fluorescence unit intensity (FU) and x axis is migration time in seconds (s) and nucleotides (nt).

Previously, we reported that LIM1863 cells release two EV subtypes – exosomes and shed microvesicles [4], and that within the exosome subtype there are two distinct exosome populations, one enriched for apical surface sorting proteins (EpCAM-Exos), the other, basolateral surface sorting protein (A33-Exos); all three EV populations have distinct proteome profiles [4]. To assess whether RNAs are specifically sorted into EVs, and whether the repertoires of miRNAs (miRs) in the three populations we isolated differ, we embarked on a large-scale purification of EVs from LIM1863 culture medium (CM). To generate enough EVs for total RNA analysis we employed a continuous cell culture approach using CELLine CL-1000 Bioreactor flasks to generate ∼1200 mL LIM1863 CM. sMVs were purified using differential centrifugation and A33-Exos and EpCAM-Exos by sequential immunoaffinity capture, see **Figure. 1A**. The exosome populations (A33-Exos and EpCAM-Exos) could not be distinguished by electron microscopy (50–150 nm diameter) whereas the sMVs were more heterogeneous in size (100–1,500 nm diameter) and consistent with the known morphology (**Figure 1** **A-D**); all three EV subpopulations contained stereotypical exosome markers (TSG101, Alix, CD9) (**Figure 1E**). This approach yielded ∼20 mg of sMVs and ∼3 mg of A33- and EpCAM-Exos. To determine if LIM1863 cell EVs contain RNA, purified EVs were extracted for total RNA including the small RNA fraction using standard RNA extraction methodology. The quality and quantity of the isolated RNA was determined using an Agilent 2100 Bioanalyzer (**Figure 1F**). The RNA yield: A33-Exos 8.8 µg RNA/∼3 mg protein, EpCAM-Exos 9.2 µg RNA/∼3 mg protein, and sMV: 72 µg RNA/∼20 mg protein. Total RNA bioanalyzer profiles indicated that LIM1863 cell-derived sMVs contained 18S and 28S ribosomal RNA (rRNA) whereas A33- and EpCAM-Exos lack detectable amounts of these species of RNA in agreement with exosomes derived from other cell lines [22], [38], [39], [40].

### A snapshot of small RNA sequencing data

To characterize small RNAs in LIM1863-derived EVs, Illumina HiSeq 2000 high-throughput technology was employed to sequence four small RNA libraries (LIM1863 cells (CL), sMVs, A33-Exos and EpCAM-Exos). Initially, 20330356, 25388242, 22512338, and 24096270 raw reads were produced. After trimming low-quality reads, adaptor sequences and reads where lengths were smaller than 18 nt (BGI in-house software), corresponding 18850584, 22762038, 16407260 and 18195289 total clean reads were obtained. We next mapped all clean reads to miRBase (v.20) to annotate known miRNAs in each library. The results showed 15367876, 152815949, 12771308, and 13611284 annotated clean reads corresponding to CL, sMVs, A33-Exos, and EpCAM-Exos, respectively; clean reads identified for other small RNA categories (rRNA, tRNA, snRNA, snoRNA, srpRNA, repeat-associated RNAs, mRNA degradation) and unannotated RNAs are shown in **Table 1**. The percentage of miRNAs in the total RNA isolated from each sample corresponded to 77.84, 74.81, 67.14 and 81.52 for A33-Exos, EpCAM-Exos, sMVs, and CL, respectively.

**Table 1:** Summary of small RNA sequencing of LIM1863 cell and extracellular vesicles.

	A33-Exos		EpCAM-Exos		sMVs		CL	
Raw reads	22512338		24096270		25388242		20330356	
Clean reads^a^	16407260	72.88%	18195289	75.51%	22762038	89.66%	18850584	92.72%
Mapping to genome^b^	14413370	87.85%	15436568	84.84%	20213639	88.80%	16417491	87.09%
Mapping to miRBase^b^	12771308	77.84%	13611284	74.81%	15281549	67.14%	15367876	81.52%
rRNA etc. (rRNA, tRNA, snRNA, snoRNA, srpRNA)^b^	1634937	9.96%	1721537	9.46%	4857190	21.34%	879429	4.67%
Repeat associated RNAs^b^	13778	0.08%	24616	0.14%	24383	0.11%	33957	0.18%
mRNA degraded fragments^b^	79126	0.48%	161298	0.89%	159757	0.70%	132894	0.70%
Unannotated^b^	1908111	11.63%	2676554	14.71%	2419159	10.63%	2436428	12.92%

a calculated as a percentage of raw reads.

b calculated as a percentage of clean reads.

We next examined the four LIM1863 cell-derived miRNA libraries to ascertain how many of the 2578 known miRNAs in miRBase v20 were detectable. Without a cut-off 891, 863, 770, and 759 miRNAs were represented in CL, sMVs, A33-Exos and EpCAM-Exos, respectively. However, for this study, we decided to use a more stringent threshold (>5 TPM cut-off) to allow us to focus on highly-represented miRNAs. This resulted in a total of 254 miRNAs for further analysis (**Table S1**), including hierarchical clustering of expression levels (**Figure S1**).

### LIM1863-derived EVs contain 254 distinct miRNAs

**Figure 2:**
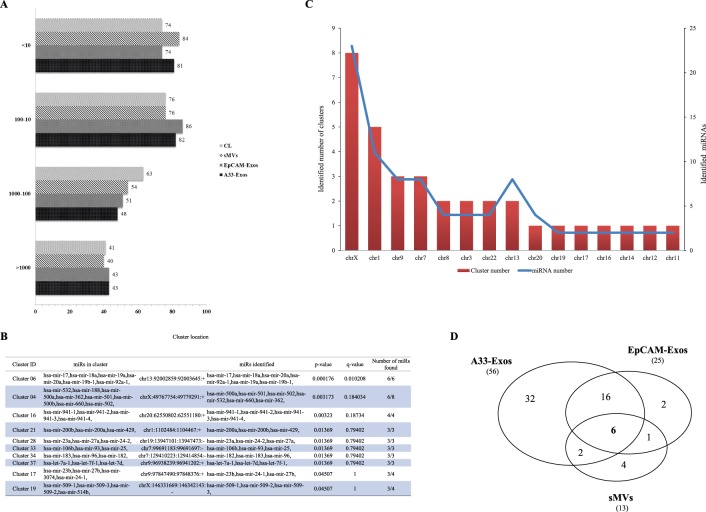
Characterisation of 254 highly-represented miRNAs in all four miRNA libraries (A33-Exos, EpCAM-Exos, sMVs and CL). (**A**) Distribution of known miRNA sequences in CL, sMVs, A33- and EpCAM-Exos based on normalised expression values (transcripts per million reads, TPM). (**B**) Top 10 miRNA clusters based on analysis of the 254 miRNAs identified with respect to their location on the human reference genome (hg19). (**C**) Distribution of clustered miRNAs on different human chromosomes. Clustered miRNAs (including miRNA number) were identified according to their chromosomal locations, which should be within 10 k bp on the chromosome; miRNAs from the same precursor (-5p, -3p) were only considered once. (**D**) Three-way Venn diagram depicting the 63 miRNAs enriched in sMVs, A33- and EpCAM-Exos, relative to CL. 6 miRNAs are common to all EVs, while 22 miRNAs are common to both exosomal datasets. miRNAS selectively enriched in each EV miRNA dataset: A33-Exos 32/56, EpCAM-Exos 2/25, and sMVs 4/13.

An inspection of **Table S1** shows that the 254 miRNAs are represented in all four libraries, albeit at varying levels of enrichment. Significantly, more than 75% of these 254 miRNAs are highly represented in each library (>10 TPM) (**Figure 2A**), and the top 20 miRNAs in the A33-Exos, EpCAM-Exos, sMVs and CL libraries represent 91.02%, 90.72%, 91.02%, and 91.42% of the corresponding total reads of miRNAs. Interestingly, the top three most highly-represented miRNAs identified in LIM1863-derived EVs - miR-192-5p, miR-10a-5p, and miR-191-5p - have been reported previously in tissue and serum of CRC patients as potential diagnostic biomarkers. For example, miR-192 has been observed in tissue [41] and serum/plasma [42] from CRC patients, miR-191 in tissue [41], [43] and serum/plasma [44], and miR-10a in tissue [45] and serum/plasma [42] from CRC patients. Moreover, miR-192 is reported to suppress metastasis of CRC [46] and its synthesis, along with that of miR-215 (also highly represented in our 254 miRNA dataset), is induced by p53 and shown to play an important regulatory role of genes involved in the TGF-β signalling pathway [47], [48].

We next performed a detailed miRNA cluster analysis (i.e., identifying groups of miRNAs encoded by polycistronic transcripts, thought to be co-expressed [49]) of the 254 miRNA datasets. Of the 153 known miRNA clusters located within 10 K bp distance on the human genome 34 were identified. Several of these clusters are statistically enriched (**Figure 2B**, **Table S2**). According to *p*-values calculated by Fisher’s exact test the top five miRNA clusters identified are *cluster 6* (6/6 members identified; miR-17, -18a, -19a, -19b-1, -20a, and -92a-1), *cluster 4* (6/8 members identified; miR-532, -188, -500a, -362, -501, -500b, -660, and -502), *cluster 16* (4/4, miR-941-1, -941-2, -941-3, and 941-4), *cluster 21* (3/3 members identified; miR-200a/200b/429), and *cluster 28* (3/3 members identified; miR-23a, -27a, and 24-2). Of these, *cluster 6* miRNAs (miR-17∼92a family) have been reported to be highly represented in several solid tumours including breast, lung, colon, prostate and stomach [50]. Expression of *cluster 4* miRNAs are regulated by E2F1 [50], can directly target BCL2/11/Bim to supress Myc-induced B cell lymphoma genesis [51] and regulate several genes in the TGF-β pathway [52]. The chromosomal distribution of these clusters (**Figure 2C**) showed chromosomes-X (8 clusters), −1 (5 clusters), and −9 (3 clusters) to be the most prominent.

We next examined the representation of various miRNA family members in the 254 miRNA dataset (**Table S3**). This analysis revealed the prominence in all three EVs of members from the following families: let-7 (12/12 members observed, let-7a/b/c/d/e/f/g/i, miR-98-5p), miR-181 (6/6, miR-181a-1/a-2/b-1/b-2/c/d), miR-30 (6/6, miR-30a/b/c-1/c-2/d/e), miR-320 (7/8, miR-320a/b-1/b-2/c-1/c-2/d-1/d-2), miR-8 (5/5, miR-141, miR-200a/b/c and miR-429), miR-17 (6/8, miR-106a/b, miR-17, miR-18a, miR-20a and miR-93), miR-192 (2/2, miR-192 and miR-215) and miR-25 (3/4, mir-25 and mir-92a/b).

### 63 miRNAs are preferentially enriched in LIM1863-derived EVs

To assess whether some miRNAs are specifically sorted into EVs we conducted a miRNA-enrichment analysis for A33-Exos, EpCAM-Exos and sMVs. MiRNAs with <2 fold changes relative to CL miRNAs were filtered out, yielding 63 miRNAs for comparison (**Table 2**). MiRNA representation was most prominent in purified A33-Exos (56 miRNAs represented, of which 32 are selectively enriched compared to other EVs), followed by EpCAM-Exos (25 miRNAs, 2 selectively enriched) and sMVs (13 miRNAs, 4 selectively enriched) (**Table 2**, **Figure 2D**). There are only 6 miRNA sequences common to all three EV subtypes, including three ‘passenger strand’ miRNAs (miRNA* sequences) (miR-451a, miR-4454, miR-7641, let-7a-3p*, let-7f-1-3p* and miR-574-5p*). To date, only miRNA-451a has previously been observed in EVs (embryonic stem cell-derived EVs, [53]). The significance of three miRNA* sequences being common to all three EV subtypes is not clear at this stage and must await analysis of a statistically significant number of EV samples derived from other CRC cell line sources. Overall, in the enriched 63 miRNA dataset we observe 12 certain miRNA* sequences, three of which (let-7a-3p*, let-7f-1-3p*, miR-574-5p*), are highly represented in all EV subpopulations (**Table 2**).

**Table 2:** miRNAs enriched in different EV subtypes.

	miR ID	A33-Exos	EpCAM-Exos	sMVs	CL	log_2_ (A33-Exos/CL)	log_2_ (EpCAM-Exos/CL)	log_2_ (sMVs/CL)	log_2_ (A33-/EpCAM-Exos)	log_2_ (Exos/sMV)	Colorectal Cancer Association^a^
**Only A33-Exos**	hsa-miR-3677-3p	5.06	0.55	0.13	0.48	3.41	0.20	−1.86	3.20	4.33	
	hsa-miR-362-3p*^+^	10.79	1.92	1.14	1.96	2.46	−0.03	−0.78	2.49	2.42	Y
	hsa-miR-19b-3p	11230.33	2816.61	3876.76	2093.94	2.42	0.43	0.89	2.00	0.81	Y
	hsa-miR-425-3p*	81.61	27.53	25.26	15.97	2.35	0.79	0.66	1.57	1.07	Y
	hsa-miR-19a-3p	2550.64	603.95	887.84	585.50	2.12	0.04	0.60	2.08	0.78	Y
	hsa-miR-378c	569.08	152.13	169.49	140.63	2.02	0.11	0.27	1.90	1.05	Y
	hsa-miR-192-3p*	23.04	11.16	4.88	5.99	1.94	0.90	−0.30	1.05	1.78	Y
	hsa-miR-3613-3p*^+^	8.78	4.07	1.58	2.33	1.91	0.80	−0.56	1.11	1.99	
	hsa-miR-378d	30.11	5.99	8.57	8.65	1.80	−0.53	−0.01	2.33	1.02	Y
	hsa-miR-652-3p	43.94	17.97	8.08	12.63	1.80	0.51	−0.64	1.29	1.91	
	hsa-miR-331-5p	10.73	4.18	3.30	3.13	1.78	0.42	0.07	1.36	1.14	
	hsa-miR-3664-3p	5.06	2.64	1.98	1.59	1.67	0.73	0.31	0.94	0.93	
	hsa-miR-107	2272.59	1135.90	1548.19	813.13	1.48	0.48	0.93	1.00	0.11	Y
	hsa-miR-23a-3p	330.59	186.70	91.42	119.89	1.46	0.64	−0.39	0.82	1.48	Y
	hsa-miR-130 b-3p	416.83	163.28	181.05	158.83	1.39	0.04	0.19	1.35	0.65	Y
	hsa-miR-10a-3p*	155.85	86.56	50.70	60.69	1.36	0.51	−0.26	0.85	1.24	
	hsa-miR-3200-3p	6.09	3.41	3.34	2.39	1.35	0.51	0.48	0.84	0.49	
	hsa-miR-140-3p	185.34	97.72	91.86	73.00	1.34	0.42	0.33	0.92	0.60	
	hsa-miR-423-5p	6932.05	2301.26	1271.59	2787.18	1.31	−0.28	−1.13	1.59	1.82	
	$hsa-miR-106b-3p*^+^	351.00	261.61	168.17	144.56	1.28	0.86	0.22	0.42	0.85	
	hsa-miR-15b-3p*	28.16	16.93	14.45	11.78	1.26	0.52	0.30	0.73	0.62	
	hsa-miR-629-5p	48.58	20.44	23.59	20.32	1.26	0.01	0.22	1.25	0.52	Y
	hsa-miR-378a-3p	9392.79	4318.98	4269.65	4001.47	1.23	0.11	0.09	1.12	0.66	Y
	hsa-miR-584-5p	881.44	287.16	306.83	387.57	1.19	-0.43	−0.34	1.62	0.89	Y
	hsa-miR-203b-3p	19.38	13.47	14.23	8.54	1.18	0.66	0.74	0.53	0.19	Y
	hsa-miR-1307-5p*	42.05	9.45	5.36	18.89	1.15	−1.00	−1.82	2.15	2.22	
	hsa-miR-502-3p	68.38	33.14	26.14	30.77	1.15	0.11	−0.24	1.05	0.93	
	hsa-miR-143-3p	7.80	6.21	4.70	3.66	1.09	0.76	0.36	0.33	0.56	Y
	hsa-miR-93-3p*	6.58	2.25	1.45	3.13	1.07	−0.47	−1.11	1.55	1.56	
	hsa-miR-532-5p	1241.95	952.83	905.94	603.48	1.04	0.66	0.59	0.38	0.27	Y
	hsa-miR-365a-3p	18.53	10.11	9.14	9.07	1.03	0.16	0.01	0.87	0.63	Y
	hsa-miR-365b-3p	18.53	10.11	9.14	9.07	1.03	0.16	0.01	0.87	0.63	Y
**Only EpCAM-Exos**	hsa-miR-106a-5p	1.16	6.49	1.23	0.80	0.54	3.03	0.63	−2.49	1.68	Y
	hsa-miR-577	1075.19	3040.29	1702.18	1114.77	-0.05	1.45	0.61	−1.50	0.31	Y
**Only sMVs**	hsa-miR-675-5p	3.60	0.93	11.07	1.86	0.95	−0.99	2.58	1.94	−2.33	Y
	hsa-miR-7704	1.46	0.93	5.23	1.11	0.39	−0.25	2.23	0.65	−2.18	
	hsa-miR-98-5p	45.77	294.25	1310.52	358.87	−2.97	−0.29	1.87	−2.68	−2.89	Y
	hsa-miR-664a-3p	12.86	16.27	20.91	8.28	0.64	0.98	1.34	−0.34	−0.52	
**All EVs**	hsa-miR-4454	7.25	1.87	1.01	0.32	4.51	2.55	1.67	1.96	2.12	
	hsa-let-7f-1-3p*	14.87	5.44	5.01	0.95	3.96	2.51	2.39	1.45	0.98	
	hsa-let-7a-3p*	39.07	21.65	10.41	5.04	2.95	2.10	1.05	0.85	1.52	
	hsa-miR-574-5p*^+^	39.86	16.54	42.75	6.21	2.68	1.41	2.78	1.27	−0.63	
	hsa-miR-451a	2.62	2.86	10.32	1.06	1.30	1.43	3.28	−0.12	−1.93	Y
	hsa-miR-7641	15.66	67.65	123.23	7.64	1.04	3.15	4.01	−2.11	−1.52	
**Both A33-/EpCAM-Exos**	hsa-miR-320d	547.99	97.22	15.11	7.80	6.13	3.64	0.95	2.49	4.36	Y
	hsa-miR-320c	609.12	116.84	21.44	11.56	5.72	3.34	0.89	2.38	4.03	Y
	hsa-let-7i-3p*	5.24	3.52	0.40	0.37	3.82	3.24	0.09	0.58	3.45	
	hsa-miR-374b-5p	352.41	113.22	37.52	25.52	3.79	2.15	0.56	1.64	2.59	
	hsa-miR-320b	2227.92	802.30	246.16	327.52	2.77	1.29	−0.41	1.47	2.59	Y
	hsa-miR-4664-3p	5.36	2.75	0.62	0.95	2.49	1.52	−0.63	0.96	2.70	
	hsa-miR-320a	3801.67	1659.61	726.65	780.45	2.28	1.09	−0.10	1.20	1.88	Y
	hsa-miR-221-3p	2624.33	1289.18	821.63	579.13	2.18	1.15	0.50	1.03	1.23	Y
	hsa-miR-1266-5p	8.78	5.61	2.64	2.49	1.82	1.17	0.08	0.65	1.43	
	hsa-miR-576-3p	5.67	5.77	3.08	1.91	1.57	1.60	0.69	−0.03	0.90	
	hsa-miR-549a	33.58	27.86	16.74	11.35	1.56	1.30	0.56	0.27	0.87	Y
	hsa-miR-1246	348.57	307.55	168.17	126.42	1.46	1.28	0.41	0.18	0.96	
	hsa-miR-3605-5p	77.10	58.37	38.53	29.07	1.41	1.01	0.41	0.40	0.80	
	hsa-miR-200c-3p	1084.09	1430.10	563.31	477.97	1.18	1.58	0.24	−0.40	1.17	Y
	hsa-miR-95-3p	18.96	29.24	13.66	8.38	1.18	1.80	0.70	−0.63	0.83	Y
	hsa-miR-146a-5p	230.81	234.18	178.59	107.95	1.10	1.12	0.73	−0.02	0.38	Y
**Both A33-Exos/sMVs**	hsa-miR-193a-3p	22.12	8.02	46.92	6.58	1.75	0.29	2.83	1.46	−1.68	Y
	hsa-miR-203a	1717.84	1258.57	1475.97	728.62	1.24	0.79	1.02	0.45	0.00	Y
**Both EpCAM-Exos/sMVs**	hsa-miR-204-5p	45.41	93.71	96.13	27.64	0.72	1.76	1.80	−1.05	−0.44	Y

a miRNA data for human CRC tissue/biofluid [65], [91], [92], [94], [97], [98].

* miRNA star sequences identified in this study, according to miRBase.

+ Enriched miRNA star sequence in EV in comparison to mature miRNA.

$ miRNA star sequence expressed greater than the mature miRNA in all four libraries.

We next examined the 63 miRNA dataset (**Table 2**) to ascertain whether there were any miRNAs that enabled distinction between exosomes (A33-Exos and EpCAM-Exos) and sMVs. This analysis revealed 7 miRNAs significantly enriched in exosomes (hsa-miR-320a 320b, -320c, -320d 221-3p, -374-5p, and -200c-3p), compared to sMVs. Strikingly, miRs-320a/b, -221-3p and -200c-3pc had more than 1000 TPM in each exosome library with 1.18–2.28 log_2_ ratio fold changes compared to corresponding values in the range 500–800 TPM (−0.33–1.88 log_2_ fold changes) in sMVs and CL libraries. MiR-320a is implicated in CRC due to its ability to suppress cell proliferation by targeting β-catenin [54]. The expression of miR-320a can be used to evaluate the risk of CRC metastasis due to its ability to bind directly to the 3′-UTR of neurophilin (NRP-1), a co-receptor of vascular epithelial growth factor [55]. The presence of miR-320a in plasma has been reported as a potential biomarker for the early detection of CRC [44]. miR-221-3p, along with miR-222 which we also see in the highly-expressed 254 miRNA dataset (**Table S1**), has been validated experimentally to regulate cell proliferation by targeting p27/Kip1, a cell cycle inhibitor and tumour suppressor, to promote tumourigenesis [56], [57]. Interestingly, elevated levels of miR-222, along with miR-17-3p, -135b, -92 and -95, have been reported in CRC patient plasma and tumour tissue [58]. miR-200c,which along with miR-141 is a member of the miR-141∼200c cluster (cluster 59, **Table S2**) as well as the miR-8 family (**Table S3**) targets the transcriptional repressor zinc-finger E-box binding homeobox 1/2 (ZEB1/2) [59] and SIP1 [60] is a critical inducer of EMT in several cancer types, including colorectal [61]. Circulating miR-141 is a potential biomarker for CRC metastasis [62].

A salient feature of the 63 miRNAs (TPM >5) enriched in LIM1863-derived EVs (**Table 2**) was the observation that 38 were exclusively represented in *one* or *other* of the three EV libraries, relative to the CL library. Foremost, was the finding that 32/38 identified miRNAs were exclusive to A33-Exos. Of these, miRs-19a-3p, -19b-3p, the miR-378 family (miRs-378a-3p, -378c and -378d), -107 and the miR-320a/b predominate. Although the miRs-320a/b are also enriched in EpCAM-Exos, they are more enriched in A33-Exos, especially miR-320a (TPM value 3801.67, 2.28 log_2_ ratio fold change relative to CL representation). miRNA-107 is also exclusively represented in A33-Exos, albeit to a lesser extent than miRs-19a/b, -378a/b/cd and -320a/b. miRs-19a/b are key oncogenic miRNAs from the miR-17∼92a cluster [63], and implicated in various cancers and reported to regulate gene expression levels of important cancer pathways and immune modulatory systems [64]. It is thought that miRs-19a/b are induced by Myc, and regulate cell survival by targeting the expression of PTEN [20], Bcl2L11, Prkaa1 and PP2A [64]; both miRs-19a/b have been observed in CRC patient tissue and blood and reported to be potential biomarkers for this disease [42], [65]. miRs-378a/b/c/d are reported to influence cell survival, tumour growth and angiogenesis by targeting the expression of SuFu and Fus-1 expression [66]. miRs-320c/d are reported to inhibit cell proliferation by targeting the transferrin receptor 1 (CD71) [67]; in the case of CRC they are reported to suppress proliferation by targeting β-catenin and the Wnt-signalling pathway [54]. MiR-107, which is induced by p53, is reported to inhibit HIF-1 and thereby tumour angiogenesis [68]. Along with miR-103, miR-107 can promote CRC metastasis by targeting the metastatic suppressors DAPK and KLF4 [69].

Inspection of **Table 2** also reveals that EpCAM-Exos and sMVs also contain miRNAs exclusively enriched relative to A33-Exos. For example, EpCAM-Exos are significantly enriched with miR-577 (TPM 3040.29 compared to 1075.19 and 1702.18 for A33-Exos and sMVs, respectively) and sMVs contain miR-98-5p (TPM 1310.52) which discriminates these vesicles from A33- and EpCAM-Exos (2.89 log_2_ fold ratio when compared to both exosome populations) (**Table 2**).

As LIM1863-derived EVs contain several proteins with an innate immunity role [4] (HMGB2/3, high-mobility group box proteins; ANPEP, alanyl aminopeptidase; TF, transferrin; LGALS3BP, lectin galactoside-binding soluble 3 binding protein; LYZ, lysozyme; LTF, lactotransferrin), we examined our miRNA datasets for the presence of miRNAs reported to be involved in the immune response [70]. **Table S4** shows members of the miR-17∼92 cluster to be highly represented in A33-Exos and, to a lesser extent, miR-106b-3p. The miR-17∼92 cluster has been reported to regulate B- and T-cell development by targeting Bim/PTEN [51], [71], and miR-106b-3p is implicated in the control of monocytopoiesis [72].

### qRT-PCR validation for EV-enriched miRNAs

**Figure 3:**
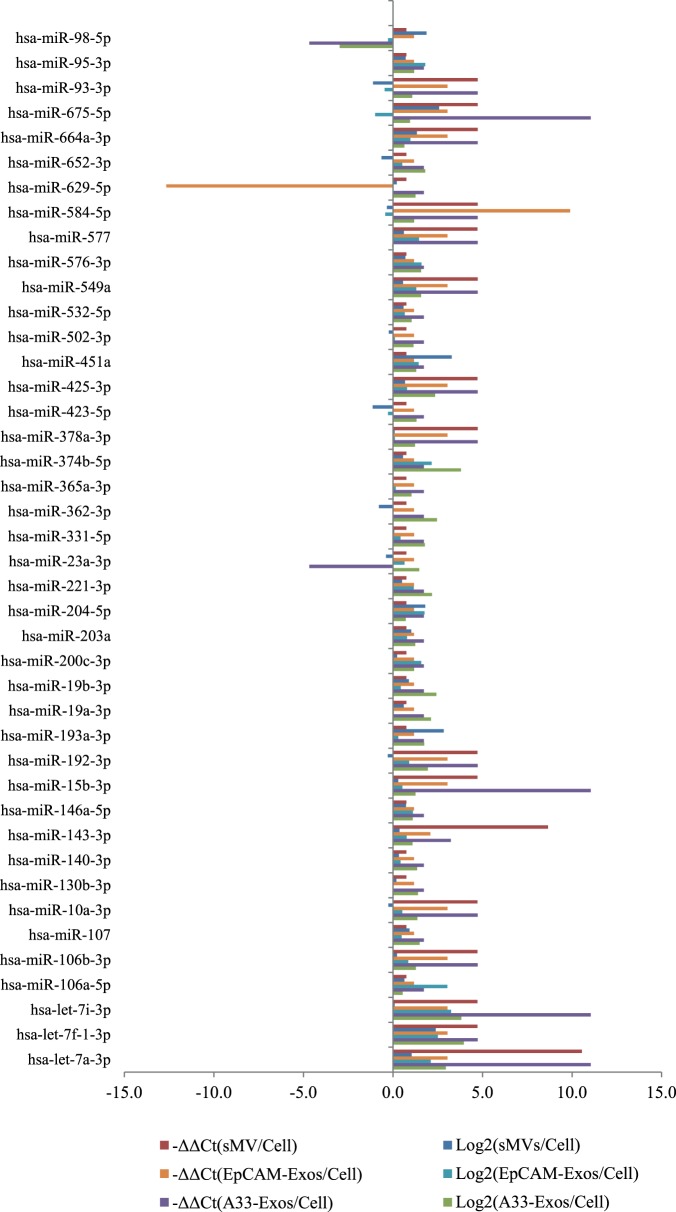
EV-enriched miRNAs from qRT-PCR and Deep Sequencing Data. The normalized expression values were log_2_ (deep sequencing) and −ΔΔCt (qRT-PCR) transformed. Comparisons between A33-Exos/CL, EpCAM-Exos/CL, and sMVs/CL are shown.

To validate the miRNA expression changes identified by Illumina HiSeq 2000 platform, we performed qRT-PCR using TaqMan array cards A+B (set v3.0) representing 754 assays specific to human miRNAs (miRBase v14). As shown in **Figure 3**, 42 of the 63 (66.7%) highly enriched miRNAs, seen in our study were identified – the discrepancy being due to the higher coverage of miRNAs in miRBase v20 used in our Illumina HiSeq studies. Overall, 33/42 (78.6%) miRNAs detected by qRT-PCR were consistent in expression with the deep sequencing results. For individual EV datasets with similar expression trends for qRT-PCR validation, 40 miRNAs (95.2%) were observed in A33-Exos, 35 (83.3%) for EpCAM-Exos, while 33 (78.6%) miRNAs were identified with same expression patterns in sMVs.

### Gene ontology (GO) and KEGG pathway enrichment analysis

To generate further insights into potential signalling pathway perturbation following EV uptake by recipient cells, we performed GO and KEGG pathway analysis. GO analysis of our enriched 63 miRNAs dataset reflects a strong correlation (21 GO annotated terms representing 10% (1265), **Figure S2**) of target genes predicted by TargetScan between the target genes of these miRNAs and proteins associated with extracellular matrix, membranes and cancer progression. KEGG pathway analysis (**Table S5**) of the 63 miRNA dataset suggests that these EV miRNAs may modulate several genes associated with signalling pathways in recipient cells – these include important pathways implicated in cancer such as the Wnt, Ras, TGF-β, and p53 signalling pathways [73], [74], [75].

## Discussion

EVs are nanometer-sized membraneous particles (30 nm to 2,000 nm in diameter) released from most cell types under both normal and pathological conditions [1], [11]. Through their diverse cargo (proteins, lipids, RNA, DNA) they play a pivotal role in intercellular communication [13], including disease pathogenesis [12] such as driving the formation of a pre-metastatic tumour niche [14], [15], [16], [17]. One of the challenges of EV research is the recognition that these extracellular vesicles are heterogeneous comprising three main subtypes – exosomes (50–150 nm), shed microvesicles (sMVs), 400–1,500 nm) and apoptotic bodies (500–2,000 nm) [2], and the technical difficulties involved in purifying them to homogeneity [3]. Recently, we examined different methods for purifying exosomes [3] and sMVs [4] from CRC cell lines. These studies led to the finding, based upon proteome profiling, that two distinct populations of exosomes (A33-Exos and EpCAM-Exos) are released from LIM1863 colon carcinoma cell-derived organoids into cell culture medium, along with sMVs, and that both exosome populations differ significantly at the protein level from sMVs [4]. Notably, the A33-Exos contained proteins consistent with release from the basolateral surface and EpCAM-Exos, from the apical cell surface. It is becoming increasingly clear that if we want to fully understand EV biology and their physiological role then they need to be studied using a combination of other *omics* data, such as lipidomics and RNA biology.

Several studies have described the expression profiles of miRNAs in EVs [22], [23], [40], [76] and, more recently, that exogenous miRNAs delivered by EVs regulate the expression of target genes and recipient cell function [27], [77], [78], [79]. In the present study, we have characterised the miRNA expression profiles in three distinct EV subtypes released from CRC cell line LIM1863. Our results show that there are 254 miRNAs in total in the three EV subtypes that are significantly enriched (>5 TPM) relative to the parental LIM1863 cells. Of these, miR-192-5p, miR-10a-5p and miR-191-5p are the most highly represented our dataset along with members of the let-7 and miR-8 families. Of these 254 miRNAs, 63 were enriched in the EVs more than 2-fold relative to our CL miRNA dataset. There are 6 miRNAs common to the three LIM1863-dervided EVs - let-7a-3p*, let-7f-1-3p*, miR-451a, miR-574-5p*, miR-4454 and miR-7641 and 6 exosome miRNAs that enable discrimination between sMVs (miR-320a/b/c/d, miR-221-3p, and miR-200c-3p); we also report one miRNA (miR-98-5p) observed in sMVs but not in A33- or EpCAM-Exos. While this is the first report to our knowledge of miRNAs that can discriminate between different extracellular vesicles, we need to be cautious about this interpretation until more homogeneous vesicle types are miRNA profiled. Notably, most of the LIM1863-dervied EV miRNAs we see in our study are observed in A33-Exos-56 compared to 25 and 13 found in EpCAM-Exos and sMVs, respectively. Of the 56 miRNAs enriched in A33-Exos, 32 are selectively enriched (when compared to EpCAM-Exos and sMVs) (**Table 2**), including 9 miRNA* sequences (miRs-425-3p*, -192-3p*, -362-3p*, -10a-3p*, -3613-3p*, -106b-3p*, -15b-3p*, -1307-5p*, and -93-3p*).

**Figure 4:**
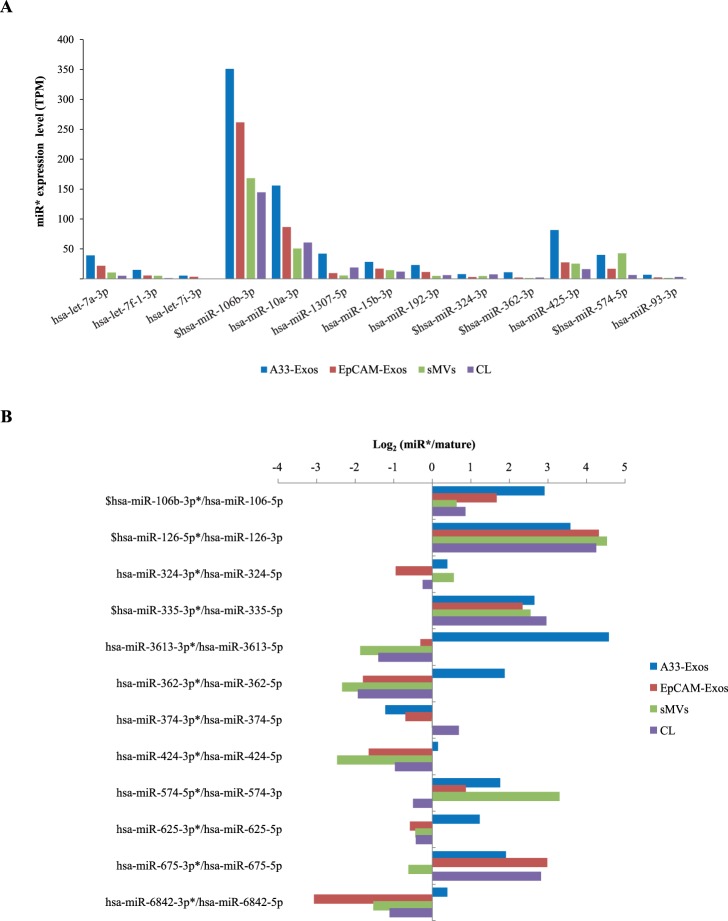
miRNA star sequences (miRNA*) are enriched in LIM1863-derived EVs. (**A**) 13 miR star sequences (miRNA*) selectively enriched in LIM1863-derived EVs, compared to CL (based on Table 2). A33-Exos (*blue*), EpCAM-Exos (*red*), sMVs (*green*), CL (*purple*). $, miR star sequences highly enriched in all four miRNA libraries. (**B**) Log_2_ values for passenger strand miRNA (miRNA*)/mature strand miRNA in all four miRNA libraries (based on 254 highly-represented miRNAs). A33-Exos (*blue*), EpCAM-Exos (*red*), sMVs (*green*), CL (*purple*). $ denotes miRNA* more highly expressed than corresponding mature miRNA in all 4 libraries; *^+^ denotes miRNA* strands selectively enriched in A33-Exos only, compared to mature miRNA strand expression.

During miRNA biogenesis, the miRNA precursor is generated in the cytoplasm as a double-stranded miRNA duplex [80] and after processing of the precursor RNA duplex there is predominant accumulation of one dominant strand, either the ‘5p’ or ‘3p’ strand, thought to be the mature functional effector miRNA while the other strand, known as the star strand (miRNA*) or ‘passenger’ strand, is degraded and typically maintained at lower levels in the cell [81]. While both mature and star strands are generated from a single primary transcript, they have different sequences and therefore target different messenger RNAs [81]. Although it is generally believed that the mature strand is the functional miRNA [82], accumulated data indicates that miRNA* can also exert regulatory effects on gene expression [81], [83]. In this study, a total of 58 miRNA* sequences were detected, of which 13 were selectively enriched in EVs (**Figure 4A**). Interestingly, expression levels of 12 miRNA* sequences (in the 254 miRNA dataset) were greater than their corresponding mature miRNAs (annotated by miRBase 20) (**Figure 4B**). Notably, miR-106b-3p*, miR-126-5p* and miR-355-3p* were detected with higher expression levels than their corresponding mature miRNAs in all four libraries. miR-106b, a member of miR-106∼25 cluster, has been shown to down regulate the expression levels of TGFBR2, SMAD2 and BMP family genes in CRC [84], miR-126-3p to suppress breast cancer metastasis [85], and miR-126-5p to inhibit the migration and invasiveness of prostate cancer cells [86]; the function of their corresponding miRNA* sequences observed in this study awaits further experimentation. In a number of cases we found miRNA* sequences to be dominant strand in A33-Exos (miR-3613-3p*, -362-3p*, -625-3p*, -6842-3p*) to that which was dominant in the parent LIM1863 cells (**Figure 4B**). This surprise finding suggests that miRNA biogenesis may be interlinked with endosomal/exosomal processing and that exosomal miRNA* sequences might affect gene expression in recipient cells in different contexts to mature miRNAs. Alternatively, exosomes may act as a vehicle for removal of miRNA star sequences from the cell in a manner akin reported for ‘protein waste management’ [87].

**Figure 5:**
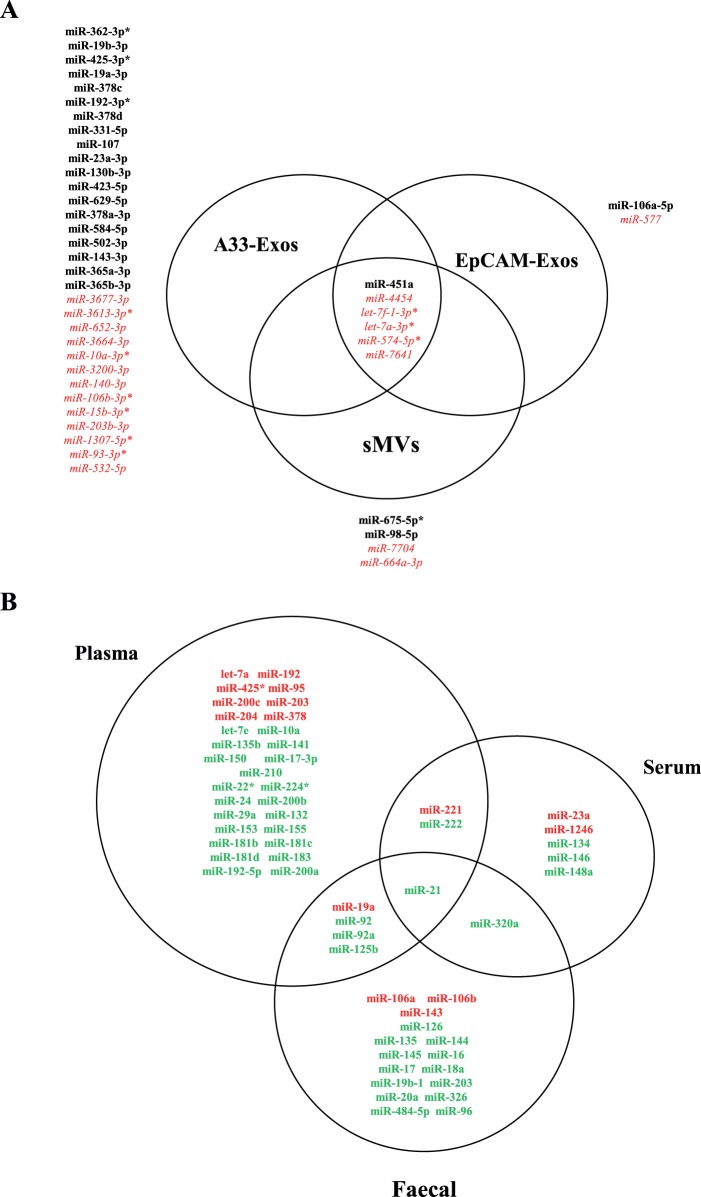
Candidate circulating miRNA biomarkers associated with extracellular vesicles and colorectal cancer. (**A**) Three-way Venn diagram depicting miRNAs identified in LIM1863-derived EVs that are associated with published miRNA data for human CRC tissue/blood and faeces [65], [91], [92], [94], [97], [98]. 6 miRNAs are common to all three EVs, while 32, 2, and 4 are selectively represented in A33-Exos, EpCAM-Exos, and sMVs, respectively. miRNAs indicated in *black* bold represent association with CRC, *red* represents miRNAs not identified in previous CRC reviews/studies. *denotes miRNA star (miRNA*sequence). (**B**) Three-way Venn diagram of identified miRNAs in EVs associated with published reports for miRNAs from human CRC plasma/serum/faecal samples human [65], [91], [92], [94], [97], [98]. Notably, miR-21 found in our study has been reported in published in human CRC plasma, serum and faeces samples. 47, 5, 16 miRNAs found in our studies have been previously reported in CRC plasma, serum, and faecal samples, respectively. *Red* represents miRNAs in our study that are highly-enriched in EVs (63 miRNA dataset); *green* represents other miRNAs identified in current study (from our 254 miRNA dataset).

Within recent years, there has been considerable interest in establishing a link between aberrant expression of miRNAs and the pathogenesis of several cancer types, including CRC [88], [89], [90], [91], [92], [93], [94]. Furthermore, there is much interest in using miRNA expression profiles as cancer classifiers [20], clinical prospective markers for early CRC detection, prognosis, prediction of disease recurrence and new pharmaceutical entities [95], [96]. In agreement with previous reports [65], [91], [92], [94], [97], [98] our findings show numerous miRNAs in the 4 libraries (A33-Exos, EpCAM-Exos, sMVs, CL) associated with CRC (**Table 2**) (for a list of oncomiRs and tumour suppresssors found in our study, see **Table S6**). Intriguingly, only 19/32 of the miRNAs enriched in A33-Exos have been previously associated with CRC according to the literature (**Figure 5A**). As miRNA expression profiles in circulating EVs have been previously reported as potential markers of disease [99], [100], [101] we examined our LIM1863-derived EV miRNA datasets for any association with previously reported miRNAs in CRC patient plasma/serum/faeces [91], [92], [94]. We detect numerous CRC-specific miRNAs previously reported in CRC patient bio-fluids in our 254- and 63-enriched miRNA datasets (**Figure 5B**). These findings suggest that the 13 selectively enriched miRNAs we observe in our A33-Exos (miR-3677-3p, -3613-3p*, 652-3p, -3664-3p, -10a-3p*,-3200-3p, -140-3p, 106b-3p*, -15b-3p*, -203-3p, -1307-5p*, -93-3p*, and miR-532-5p) that have not been previously reported in any CRC miRNA studies, to our knowledge, warrant examination as putative candidates for the development of potential CRC markers.

In conclusion, we showed significant differences between the miRNA expression profiles of three EV subtypes (two exosome populations and sMVs) secreted from LIM1863 CRC cells. Our findings provide the basis for an in-depth study, using a variety of CRC cells lines that discern the familial archetypes of CRC and accurately predict tumour microsatellite subtype, of the role of certain miRNAs as prospective diagnostic and prognostic clinical markers of this disease and offer the potential of new pharmaceutical reagents.

## Supporting Information

Figure S1 Hierarchical clustering of miRNA expression profiles of the highly expressed 254 miRNAs in cell, sMVs, and exosomes (A33-Exos and EpCAM-Exos) reveals a similarity between exosome subpopulations, and extracellular vesicles (Supplemental Table S1). CLUSTER and TREEVIEW programs were employed for hierarchical clustering and visualization of the miRNA expression profiles. Hierarchical clustering was performed with average linkage.
(PDF)

Figure S2 Gene Ontology annotation for the target genes predicted by TargetScan of the 63 miRNAs selectively enriched in LIM1863-derived EVs.
(PDF)

Table S1 Normalized expression levels of 254 miRNAs in parental LIM1863 cells (CL) and derived EVs (A33-Exos, EpCAM-Exos, sMVs).
(XLSX)

Table S2 Significant miRNA clusters characterized in LIM1863 cell and EVs.
(XLSX)

Table S3 miRNA family enrichment in LIM1863-derived EVs.
(XLSX)

Table S4 miRNAs associated with the immune response and identified in LIM1863-derived EVs.
(XLSX)

Table S5 KEGG pathway enrichment analysis for target genes of miRNAs enriched in LIM1863-derived EVs.
(XLSX)

Table S6 miRNAs from LIM1863-derived EVs and possible functional roles as oncogenes/tumour suppressors.
(XLSX)
