# Supplementary figures and images for: Deep Sequencing of RNA from Three Different Extracellular Vesicle (EV) Subtypes Released from the Human LIM1863 Colon Cancer Cell Line Uncovers Distinct Mirna-Enrichment Signatures
Source: PLoS One. 2014 Oct 17;9(10):e110314. doi: 10.1371/journal.pone.0110314 (PMC4201526; doi:10.1371/journal.pone.0110314)

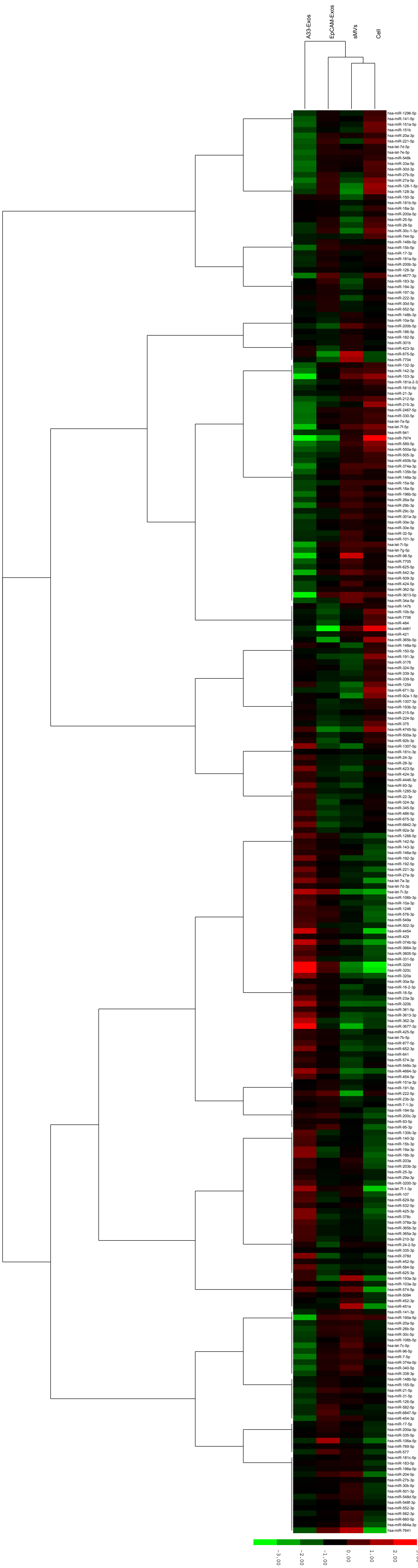

Supplement: Figure S1 — Hierarchical clustering of miRNA expression profiles of the highly expressed 254 miRNAs in cell, sMVs, and exosomes (A33-Exos and EpCAM-Exos) reveals a similarity between exosome subpopulations, and extracellular vesicles (Supplemental Table S1). CLUSTER and TREEVIEW programs were employed for hierarchical clustering and visualization of the miRNA expression profiles. Hierarchical clustering was performed with average linkage. (PDF) [file pone.0110314.s001.pdf]

Supplementary Figure S2

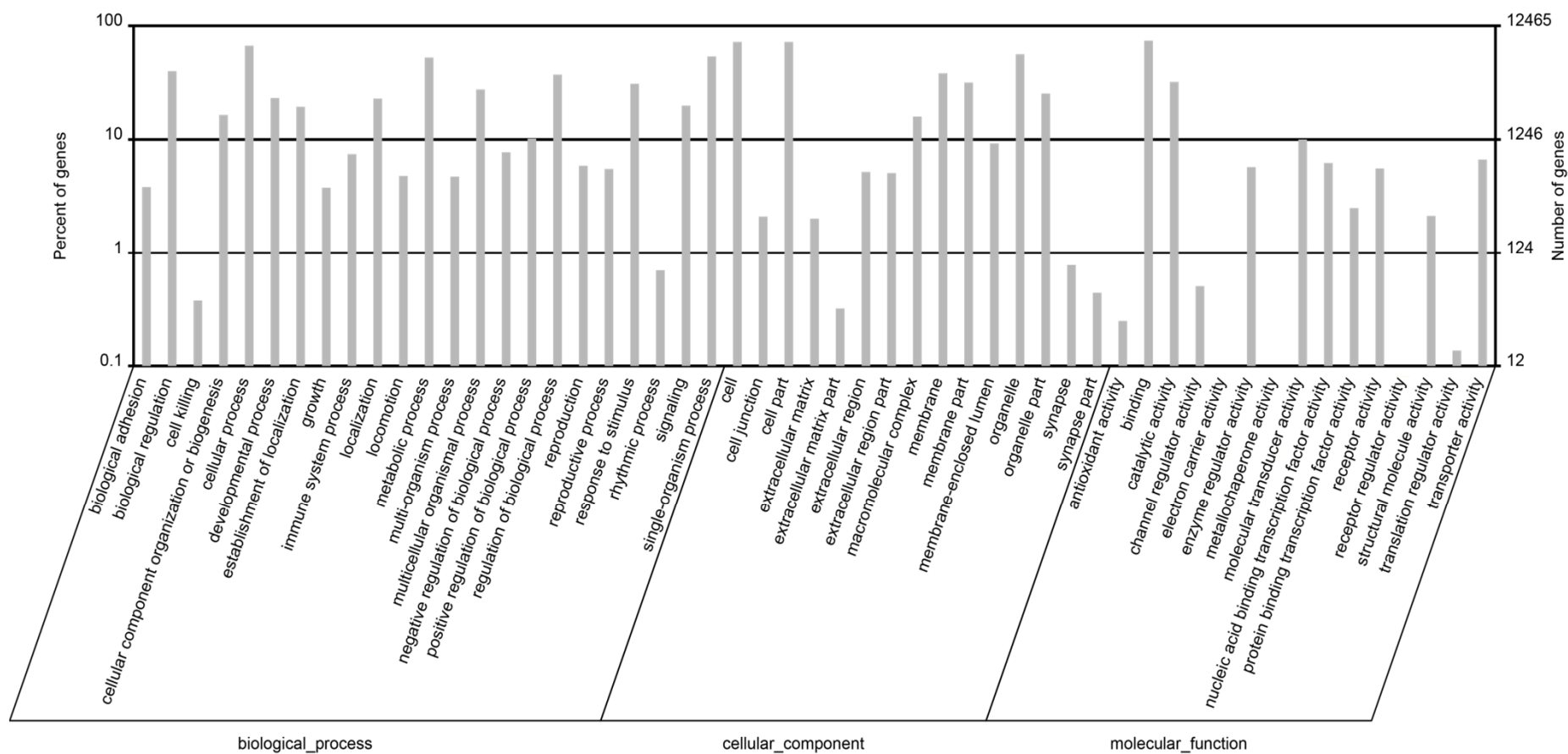

Supplement: Figure S2 — Gene Ontology annotation for the target genes predicted by TargetScan of the 63 miRNAs selectively enriched in LIM1863-derived EVs. (PDF) [file pone.0110314.s002.pdf]
